# Sleep and the Gut–Brain Axis: Bidirectional, Converging Mechanisms Linking Brain Health, Neuroinflammation, and Neurodegeneration

**DOI:** 10.3390/pathophysiology33040074

**Published:** 2026-09-30

**Authors:** Bryce Bowler, Abdul Rehman Nasir, Aldona Szewczyk, Sarah Ocanas, Archana Unnikrishnan, Claire Delpirou Nouh

**Affiliations:** 1 College of Medicine, Oklahoma University Health Center, Oklahoma City, OK 73104, USA; bryce-bowler@ou.edu; 2 Behavioral Neurology-Neurology Department, Oklahoma University Health Center, Oklahoma City, OK 73104, USA; abdulrehman-nasir@ou.edu; 3 Genes and Human Disease Research Program, Oklahoma Medical Research Foundation, Oklahoma City, OK 73104, USA; aldona-szewczyk@omrf.org (A.S.); sarah-ocanas@omrf.org (S.O.); 4 Department of Biochemistry & Physiology, Oklahoma University Health Center, Oklahoma City, OK 73104, USA; archana-unnikrishnan@ou.edu; 5 Neuroscience Program, Oklahoma University Health Center, Oklahoma City, OK 73104, USA

**Keywords:** gut–brain axis, sleep, microbiome, glymphatic system, neurodegeneration, Alzheimer’s disease, Parkinson’s disease, estrobolome, menopause, vagus nerve

## Abstract

Sleep and the gastrointestinal (GI) tract interact in a complex and reciprocal relationship, although the molecular and physiological pathways underlying this interaction remain only partially defined. Communication between the central (CNS) and enteric nervous systems (ENS) spans neural, immune, endocrine, and metabolic pathways, including vagal nerve signaling, the hypothalamic–pituitary–adrenal (HPA) axis, gut-resident microbiota, and circulating microbial metabolites, such as bile acids with neuroinflammatory or neurotransmitter activity. In this narrative review, we synthesize evidence from animal models, observational cohorts, and intervention trials to map this bidirectional axis, focusing on several underexamined modulatory mechanisms including the interaction between the gut microbiome and the glymphatic system, the brain’s perivascular waste-clearance network, which is implicated in the removal of amyloid-β (Aβ) and tau in Alzheimer’s disease; neuroendocrine regulation and sex-related differences in gut microbiome composition and sleep patterns, which may help explain the divergent neurodegenerative disease risks between men and women; and age-related parallel trajectories of microbiome senescence and sleep fragmentation in older adults. We discuss melatonin, cortisol, estrogen, and ghrelin as endocrine mediators linking circadian biology to gut physiology, and we consider how environmental and lifestyle factors further perturb this system. These mechanisms form an integrative framework of the gut–brain–sleep axis and highlight priority directions for mechanistic and translational research, including significant nodes in this pathway where targeted interventions may help cognitive resilience and slow the neurodegenerative disease progression. More research clarifying these mechanisms may help identify the most promising targets, potentially leading to personalized, precision-medicine-based therapeutic strategies.

## 1. Introduction

Sleep is one of the most fundamental physiological processes, serving critical roles in immune regulation, metabolism, memory consolidation, synaptic plasticity, and neuronal maintenance. Sleep disturbances have become a major public health concern, affecting an estimated one-third of adults worldwide and increasing in prevalence during periods of societal stress. For example, during the SARS-CoV-2 pandemic, approximately 35% (± 5%) of individuals reported significant sleep difficulties [1], with disrupted sleep being associated with increased risk for subjective cognitive decline and dementia [2]. These observations have intensified interest in identifying modifiable biological pathways linking sleep disturbances to long-term neurological health.

In parallel, the gut microbiome has emerged as a major regulator of systemic physiology and brain function [3]. The trillions of microorganisms residing within the gastrointestinal (GI) tract influence host metabolism, immune function, endocrine signaling, and neural activity [4,5]. Alterations in microbial composition and function, referred to as dysbiosis, have been implicated in a broad range of chronic diseases, including obesity, diabetes, inflammatory bowel disease, psychiatric disorders, and neurodegenerative diseases [6]. Increasing evidence suggests that sleep and the gut microbiome are closely interconnected, with sleep disturbances altering microbial composition and microbial metabolites influencing sleep architecture, circadian rhythms, and neuroinflammatory processes [7,8,9,10].

These interactions occur through the gut–brain axis (GBA), a bidirectional communication network linking the gastrointestinal tract and the central nervous system. Communication along the GBA is mediated through multiple overlapping pathways, including autonomic and vagal nerve signaling, immune mediators, microbial metabolites, endocrine pathways, and the hypothalamic–pituitary–adrenal (HPA) axis. Through these mechanisms, signals originating in the gut can influence brain function, while neural and hormonal outputs from the brain can alter gastrointestinal physiology, intestinal permeability, and microbial composition. Among the key mediators of this communication are short-chain fatty acids (SCFAs), bile acids, tryptophan metabolites, serotonin, melatonin, cytokines, and stress hormones [11,12,13].

The GBA has gained particular attention in neurodegenerative diseases. Parkinson’s disease provides one of the strongest examples of potential gut–brain connection, with GI dysfunction often preceding motor symptoms by years or even decades [14,15]. This observation has contributed to the development of body-first models of disease, which propose that pathological processes may originate in the GI tract and subsequently spread to the central nervous system. More recently, similar concepts have begun to emerge in Alzheimer’s disease (AD), where alterations in gut microbial composition, intestinal permeability, systemic inflammation, and microbial metabolite profiles have all been associated with disease progression, extending the framework developed in Parkinson’s disease (PD) [14,16,17,18]. Although neurodegenerative disorders have historically been viewed primarily as diseases of the brain, growing evidence suggests that peripheral systems may play important roles in shaping disease susceptibility and progression.

Beyond the direct effects of sleep on neural function, increasing attention has focused on the glymphatic system, a perivascular waste-clearance network that operates most efficiently during sleep [19,20,21]. The glymphatic system facilitates the removal of metabolic waste products, including amyloid-β (Aβ) and tau proteins implicated in Alzheimer’s disease. Sleep disruption impairs glymphatic function, while glymphatic dysfunction contributes to protein accumulation and neurodegeneration. Emerging evidence suggests that the gut microbiome may influence glymphatic activity through effects on blood–brain barrier integrity, neuroinflammation, astrocyte function, and aquaporin-4 (AQP4) polarization, providing a potential mechanistic bridge between gut health, sleep quality, and neurodegenerative disease [22,23,24,25,26,27,28,29].

Several additional biological factors may further modulate this interconnected system. Aging is associated with progressive alterations in both sleep architecture and microbiome composition [30], raising the possibility that parallel age-related changes contribute to increased neurodegenerative risk. Likewise, sex hormones influence both sleep physiology and gut microbial composition [31], while microbial communities, known as the estrobolome, regulate estrogen metabolism and bioavailability [32,33]. These interactions may help explain important sex differences observed in disorders such as Alzheimer’s disease, which disproportionately affects women. Environmental exposures, diet, stress, and lifestyle behaviors further shape gut microbial communities [34,35] and influence both sleep and neuroinflammatory pathways [36,37].

**Figure 1:**
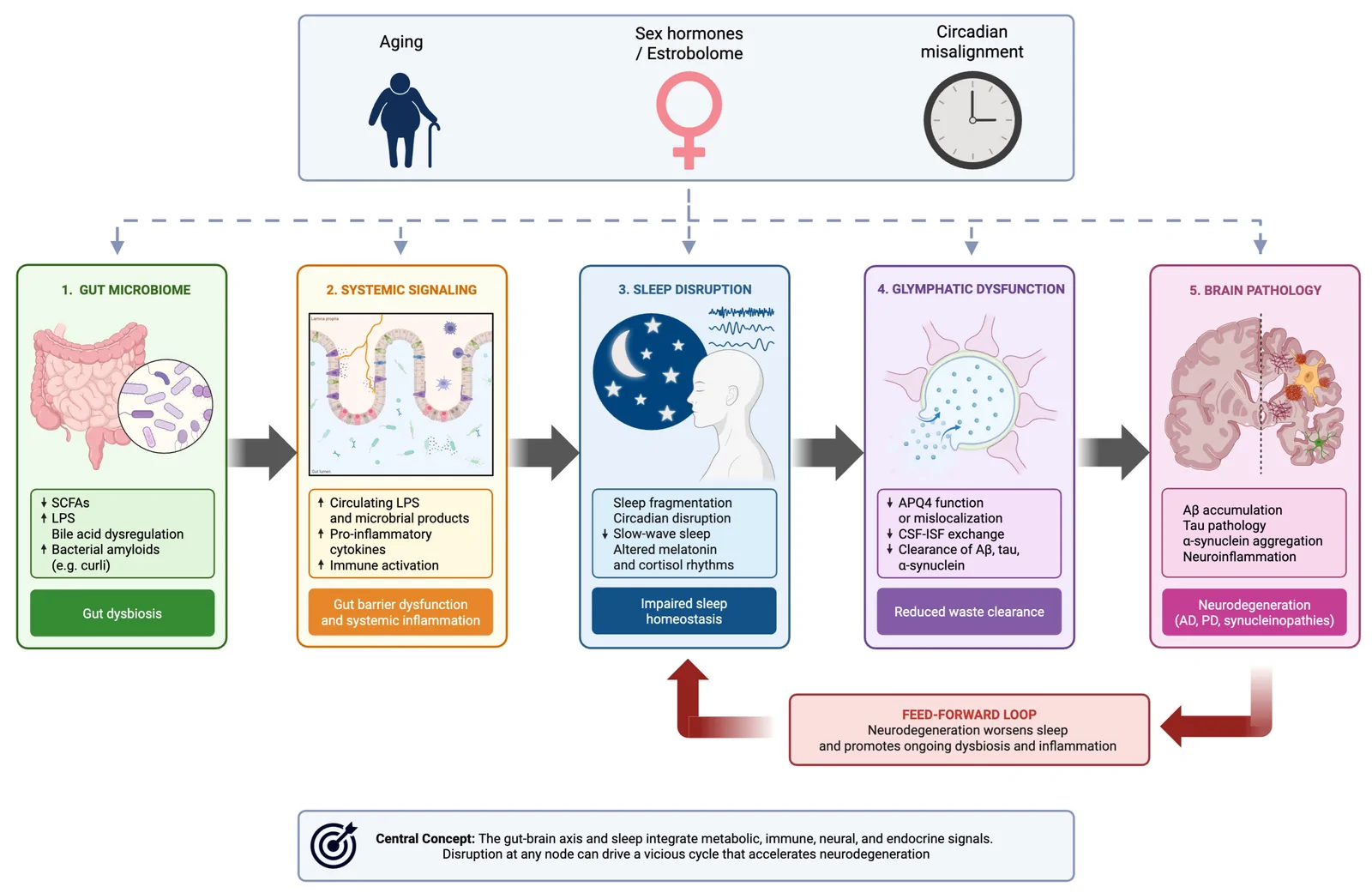
Bidirectional signaling pathways showing the interaction between gut dysbiosis, sleep disturbances and glymphatic system dysregulation. It also integrates the important role of neuroendocrine modulation via the hypothalamic–pituitary–adrenal axis and the estrobolome. Systemic translocation of microbial metabolites, as well as of pro-inflammatory cytokines and endotoxins (such as LPS), disrupts blood–brain barrier integrity, facilitating the acceleration of amyloid deposition, tau hyperphosphorylation, and the accumulation of other misfolded proteins like synuclein or TDP-43.

In this narrative review, we have aimed to distinguish evidence of this bidirectional relationship obtained from animal models, human observational or cross-sectional studies, and human intervention trials, as well as explicitly highlighting where mechanistic hypotheses currently outpace actual experimental findings. Recent reviews have explored the gut–brain–sleep relationship [38,39], the gut microbiome’s contribution to neurodegeneration [40], and the relation between insomnia and microbiota and the gut–brain axis [41]; however, other factors, including the influence of the estrobolome, sex-specific differences, and the role of the glymphatic system, remain to be integrated into this model. We explore in more detail below how sleep, gut microbial composition, endocrine signaling, immune regulation, and glymphatic clearance system function as a highly interconnected network, and how disruption at any point within this network may cascade through multiple systems, amplifying inflammation, impairing protein clearance, and ultimately increasing the vulnerability to neurodegenerative disease (Figure 1). We also look at how new biomarkers are starting to emerge, which may lead to potential diagnostic tools after further refinement. We finally end by discussing potential therapeutic opportunities that could be considered in creating personalized, multi-target treatment approaches for neurodegenerative diseases.

Sources for this review were retrieved using exclusively Google Scholar, PubMed, and the University of Oklahoma Bird Library. Due to the narrative literature review nature of this article, no strict systematic article selection algorithm was utilized to select sources. Sources were chosen based on relevance to the gut–brain–sleep network, sound methodology, and recency of publication (<10 years to present) to ensure the most updated evidence was included in this review. Note that older sources (>10 years to present) are used in some instances to support well-known and longstanding principles in the field.

## 2. Sleep, Circadian Rhythms, and the Gut Microbiome

Growing evidence indicates that sleep and circadian rhythms are major determinants of gut microbiome (GM) composition and function, highlighting a bidirectional relationship between microbial ecology and sleep physiology. Smith et al. reported that greater sleep efficiency and longer sleep duration were associated with increased GM diversity, whereas sleep fragmentation was associated with reduced diversity and altered microbial composition [7]. These observational data are further corroborated by experimental evidence suggesting that antibiotic-induced microbial depletion can trigger fragmented non-rapid eye movement sleep, indicating a bidirectional regulatory loop [17]. Chronic sleep fragmentation alters GM and fecal taxonomic profiles and has been associated with weight gain, impaired glucose tolerance, and metabolic diseases like diabetes [39,42]. Sleep-fragmentation-related GM dysbiosis may promote systemic inflammation, providing a potential mechanistic link between disturbed sleep and Alzheimer’s disease pathogenesis, as detailed later [43]. Bacterial metabolites such as short-chain fatty acids and peptidoglycans have been identified as key signaling molecules that modulate host circadian gene expression, corroborating the complexity of the microbiome–gut–brain axis [3,44]. The loss of rhythmic oscillation in the GM may indirectly predispose the central nervous system to neurodegeneration by disrupting peripheral metabolic functions [45]. These observations highlight that microbial composition and the temporal organization of microbial communities may be important for neurological health.

GM composition and function exhibit diurnal oscillations related to feeding rhythms, and modifications of these rhythms can lead to GM dysbiosis [45,46]. Disruption of these rhythmic patterns can promote dysbiosis and the expansion of pro-inflammatory or otherwise unfavorable taxa like the Coriobacteriaceae, Erysipelotrichaceae or Prevotellaceae families, while decreasing favorable ones, including the genus *Faecalibacterium* (Ruminococcaceae family) [47,48]. Gut-derived melatonin may further stabilize circadian fluctuations, acting as an endocrine bridge that maintains mucosal integrity and dampens neuroinflammatory signaling across the blood–brain barrier [49]. Modulating this molecular clock constitutes a potential therapeutic approach to stabilize these inflammatory reflexes, reducing systemic inflammation and the neuroinflammatory damage associated with neurodegenerative conditions. Beyond alterations in circadian regulation, dysbiosis can directly contribute to intestinal barrier dysfunction and chronic low-grade inflammation. Several Gram-negative bacteria, including *E. coli* and *Shigella*, have been associated with AD in both animal models and humans, displaying proinflammatory properties and contributing to leaky gut and the circulation of proinflammatory molecules [50]. GM dysbiosis leads to lipopolysaccharide (LPS) release in bloodstream leading to LPS endotoxemia and chronic smoldering inflammation [51,52].

Evidence supporting the association between sleep disruption and microbial dysbiosis has continued to accumulate in both clinical and preclinical studies. A recent systematic review and meta-analysis of human and rodent studies concluded that sleep deprivation significantly reduces alpha diversity, as measured by Shannon and Simpson indices, and increases the Firmicutes-to-Bacteroidetes ratio, a microbial pattern often correlated with metabolic dysregulation and neuroinflammatory states [8]. The disruption of gut microbial homeostasis induced by fragmented sleep has been shown to exacerbate adipose tissue inflammation and reduce insulin sensitivity, corroborating a two-hit hypothesis where sleep disturbances predispose the host to progressive neurodegenerative pathologies [17,42]. Similarly, a meta-analysis encompassing 53 studies reported decreased alpha diversity in individuals with sleep disturbances [53]. A specific pattern of GM was associated with several sleep disturbances, including insomnia, obstructive sleep apnea (OSA) and REM sleep behavioral disorder (RBD) [53]. The latter of these disturbances is particularly important considering its well-established role as a prodromal marker of synucleinopathy [54]. Additionally, reduced GM diversity was associated with OSA in a systematic review [55]. A decline in anti-inflammatory, butyrate-producing bacteria, together with greater Collinsella abundance, correlates with elevated systemic inflammatory markers and intestinal permeability seen in individuals with chronic sleep fragmentation [56,57]. These findings suggest that chronic sleep disruption promotes a pro-inflammatory microbial environment characterized by reduced microbial diversity, impaired barrier integrity, altered metabolite production, and systemic immune activation. While these findings establish a bidirectional relationship between sleep and the gut microbiome, an important question is whether these interactions contribute to the development of neurodegenerative disease. Increasing evidence suggests that disturbances in the gut–brain–sleep axis may occur years before the onset of overt neurological symptoms, leading to renewed interest in body-first models of neurodegeneration.

## 3. Brain-First and Body-First Models of Neurodegeneration

The microbiota–gut–brain axis and microbiome dysbiosis have also attracted growing interest in neurodegenerative diseases, and especially in Parkinson’s disease and other synucleinopathies, with the body-first model [14,58]. GI symptoms with constipation come early in the prodromal non-motor phase. Increased intestinal permeability in the prodromal stage has been reported [15]. In a foundational 2015 study, Scheperjans et al. reported that specific gut microbiota correlated with the Parkinson’s disease motor phenotype, including a reduced abundance of the Prevotellaceae family by 77.6% in the PD group compared with controls, and Enterobacteriaceae associated with gait difficulties and postural instability [59]. REM sleep behavior disorder happens early in the prodromal phase and RBD has been found to be associated with microbiome dysbiosis [60]. Other neurodegenerative diseases, like Alzheimer’s disease (AD), progressive supranuclear palsy (PSP), or amyotrophic lateral sclerosis (ALS), are also associated with sleep disorders and circadian rhythm dysfunction [45,61]. A growing body of evidence points to a significant role for sleep in the pathophysiology of these diseases, and more recently, a lot of attention has been placed on the glymphatic system. Recent studies have found alterations in the gut microbiome (GM) in AD, and a hypothetical mechanistic relationship between the GM and the glymphatic system [62]. New research has begun to explore whether the body-first hypothesis may extend beyond synucleinopathies to Alzheimer’s disease; however, this extension remains rather speculative, as the evidence in AD is preliminary and largely inferential compared with the more established body of evidence established with Parkinson’s disease and other synucleinopathies [63]. More recently, a similar gut-origin hypothesis has been considered for other proteinopathies, including gut-to-brain propagation of tau alongside synuclein [64], and a potential relationship between the TDP-43–immunity–microbiota axis in amyotrophic lateral sclerosis [65]. This remains theoretical, based on experimental models, and remains to be proven in humans.

For a long time, neurodegenerative diseases have been understood as brain diseases only. However, the paradigm may be slowly shifting toward a more systemic approach [63,66,67]. In alpha-synucleinopathy, Borghammer et al. proposed a dual model. In the brain-first (olfactory) subtype of the α-synuclein origin and connectome (SOC) model, α-synuclein deposition begins in the brain, possibly in the amygdala, and propagates according to the connectivity of the central nervous system, possibly unilaterally at first, up to the substantia nigra, leading to clinical motor asymmetry. In a body-first/gut-first model, the pathological process originates in the enteric nervous system before advancing to the brain via the vagus nerve to bilateral dorsal motor nuclei of the vagus, with a more bilateral clinical presentation [68,69,70,71,72]. From a gut–brain axis perspective, these two approaches may be more complementary than antagonistic. Pathology originating in the brain can disrupt gastrointestinal homeostasis through efferent top-down signaling pathways. The amygdala and locus coeruleus in particular are two important structures involved in this signaling process [73,74]. The central nucleus of the amygdala is involved in autonomic and neuroendocrine response to stress, and influences the gut via vagal modulation, impacting colonic motility and visceral sensitivity [75,76]. From the amygdala, a signal is sent to the dorsal motor nucleus of the vagus and the nucleus of the solitary tract, and overactivation of the pathway in cases of stress can increase gastric secretion and affect intestinal permeability, which creates a permissive environment for the retrograde migration of alpha-synuclein aggregates from the enteric nervous system to the brainstem [75,77]. Colonic projections from the amygdala via the locus coeruleus modulate the intestinal milieu by influencing mucosal immune responses and enteric nervous system activity. This suggests a feed-forward mechanism where central autonomic dysregulation establishes a niche for alpha-synuclein pathology to emerge in the enteric nervous system, bridging the gap between brain-first and body-first disease subtypes [70,78]. The locus coeruleus is also the main source of norepinephrine [79]. Through the modulation of sympathetic tone, this neurotransmitter system regulates intestinal transit and the integrity of the gut barrier, highlighting how the brain-first pathway can initiate or exacerbate secondary peripheral synucleinopathy [80,81]. Aberrant activity in structures like the locus coeruleus and amygdala, which govern adaptive autonomic and neuroendocrine responses, can amplify top-down signaling to the gastrointestinal tract, as seen in conditions like irritable bowel syndrome [82]. Sleep disturbances, through aberrant activation of vagal circuitry, can impair intestinal homeostasis and barrier integrity [83]. Locus coeruleus hyperactivity, often linked to visceral pathology, can cause sleep disturbances, hyperarousal, and central noradrenergic hyperactivity, leading to neuroanatomical feedback loops that may entrench systemic inflammation [84]. Stress and increased activation of the cortisol pathway may both affect sleep and stimulate locus coeruleus-mediated firing [74,85,86]. In AD, tau pathology exhibits a distinct spatial progression that aligns with anatomical connectivity, involving regions such as the locus coeruleus as an early site of involvement that precedes cortical degeneration [87]. Autonomic dysfunction has also been suggested as a possible early feature of preclinical AD, though it is underreported [88]. An autonomic dysregulation of the gastrointestinal tract can lead to dysmotility and dysbiosis, supporting the bidirectional connection.

**Figure 2:**
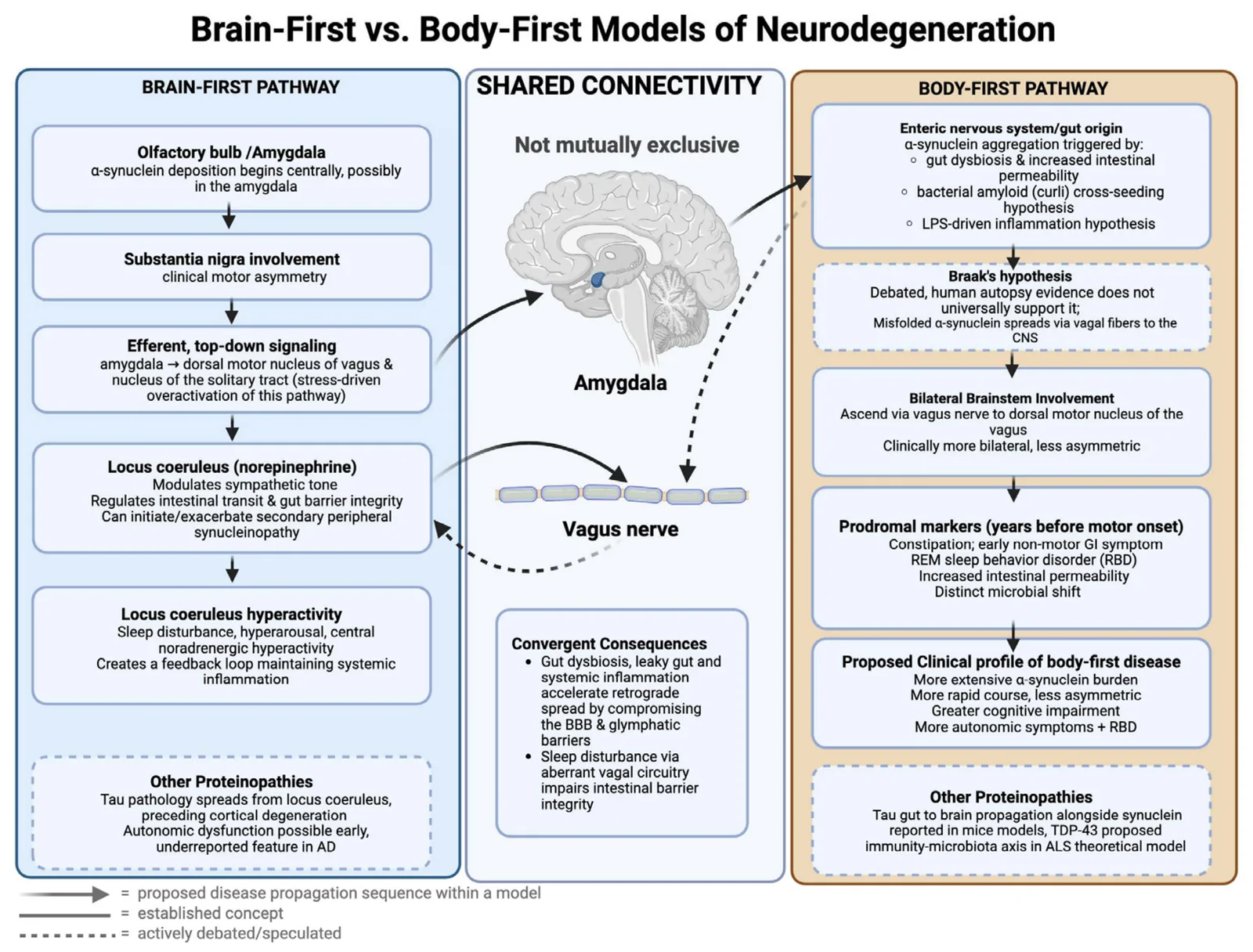
Brain-first versus body-first hypotheses. In the brain-first model (left), α-synuclein deposition begins centrally, possibly in the amygdala, with a top-down propagation. In the body-first model (right), α-synuclein originates in the periphery and propagates centripetally. Both share connectivity pathways, including through the amygdala and vagus nerve, and are not mutually exclusive (center). Similar mechanisms have been proposed for other proteinopathies, including AD and ALS. These concepts are detailed in the text and based on Borghammer et al. [68], Horsager et al. [16], Braak et al. [92], Scheperjans et al. [59] and Lionnet et al. [95]. AD: Alzheimer’s disease; ALS: amyotrophic lateral sclerosis; BBB: blood–brain barrier; CNS: central nervous system; GI: gastrointestinal; LPS: lipopolysaccharide; RBD: REM sleep behavior disorder; REM: rapid eye movement; TDP-43: TAR DNA-binding protein 43.

In the body-first model, the α-synuclein pathology burden is more extensive with a more rapid course and more significant cognitive impairment [68]. The gut-first model is also thought to be associated with more autonomic symptoms and REM sleep behavioral disorder [69]. There is still some debate around the relative prevalence of body-first versus brain-first subtypes in PD compared with dementia with Lewy bodies (DLB). Camerucci et al. did not find any significant differences between the two groups when taking the expanded criteria for the body-first definition beyond looking only at RBD before motor symptoms (to include at least two premotor symptoms among constipation, erectile dysfunction, rapid eye movement sleep behavior, anosmia, or neurogenic bladder) [89]. DLB patients also usually have more AD co-pathologies compared to PD patients [90,91]. Lastly, Braak’s hypothesis speculates that misfolded α-synuclein may originate in the enteric nervous system and spread in an antidromic manner via vagal fibers to the CNS, with gut dysbiosis, leaky gut, and systemic inflammation accelerating this process by bypassing and compromising the BBB and glymphatic barriers [92,93,94]. However, Braak’s hypothesis is still debated. Lionnet et al., 2018 concluded that human autopsy evidence does not clearly support the universal applicability of this retrograde transport model in all Parkinson’s disease cases [95]. Taken together, these observations suggest that central and peripheral mechanisms are not mutually exclusive but may interact through autonomic, immune, and neuroendocrine pathways to influence disease susceptibility and progression (Figure 2). It is likely that the gut contributes to, but is not the sole source of, neurodegenerative disease pathology. Instead, these pathologies should be viewed as complex, multifactorial syndromes where altered gut–brain communication, mediated by microbial metabolites and peripheral immune effects, synergizes with CNS-intrinsic deficits to promote neuroinflammation and synaptic dysfunction. Some underlying genetic susceptibilities or biological characteristics may add to this and potentially lower the threshold to the onset of protein aggregation and subsequent neurodegeneration to the phenotypic presentation.

The pathways discussed above are summarized in Table 1, which highlights the major biological mechanisms linking sleep, gut microbial composition, and neurodegenerative disease, along with representative biomarkers and emerging therapeutic opportunities.

## 4. Gut–Brain Communication Pathways

Understanding how pathology may spread between the gut and brain requires consideration of the biological pathways that mediate bidirectional communication between these systems. These pathways include neural, immune, endocrine, and metabolic signaling networks that are increasingly recognized as key regulators of sleep and neurodegeneration.

### 4.1. Vagal Signaling and the Propagation of Pathology

The vagus nerve (VN) occupies a central anatomical position in gut–brain communication and its complex interaction with sleep. In a study looking at VN interaction with sleep and behavior in cats, vagal nerve stimulation (VNS) was shown to significantly affect REM sleep and transition in sleep stages [96]. VNS has also been associated with increased sleep apnea events, further illustrating the complex relationship between the gut axis and sleep [97]. The vagus nerve acts as a direct path for sensory information, conveying signals from the enteric nervous system to the brainstem and may help retrograde transport of pathogens and misfolded proteins. Immune and humoral routes also contribute substantially to these processes that compromise both sleep architecture and neurological homeostasis [98]. Some microbial metabolites, such as fragilysin, by compromising the integrity of the intestinal barrier, may exacerbate this vulnerability and facilitate the translocation of neurotoxic agents that disrupt both vagal afferent signaling and restorative sleep cycles [99]. This increased intestinal permeability, or “leaky gut”, allows systemic pro-inflammatory cytokines and reactive oxygen species to exacerbate this neural dysfunction, strengthening the cycle of neurodegeneration and sleep disruption [100,101].

Vagal afferents appear to possibly streamline the transmission of misfolded proteins and inflammatory signals, potentially modulating sleep–wake architecture through their direct projections to brainstem nuclei involved in arousal regulation. In the dual dynamic brain-first versus body-first Borghammer/SOC paradigm, the vagus nerve links the brain and gut in a key bidirectional way: body-first models posit initial enteric α-synuclein aggregation with centripetal vagal spread to the brainstem, while brain-first mechanisms feature early brainstem or olfactory pathology exerting retrograde influences on gut function via efferent signaling. Truncal vagotomy in murine models has shown capacity to arrest the centripetal spread of alpha-synuclein fibrils from the enteric nervous system to these brainstem regions, which shows that the nerve plays an essential role in pathology dissemination [102]. Vagus nerve stimulation, as a potential therapeutic strategy for modulating neural activity and disease progression, is discussed in detail in the Vagus Nerve Stimulation Section [103,104]. Some clinical data indicate that the anatomical severance of vagal fibers is associated with a reduced risk of developing Parkinson’s disease. Two independent nationwide cohorts from Denmark and Sweden [105,106] have been interpreted as suggesting that interrupting these conduits may help sequester synucleinopathy within the periphery; however, this remains an epidemiological association from observational human data rather than direct causal evidence of vagal propagation [93].

Han et al. reviewed the vagus nerve, which serves as a key conduit for microbial signaling within the bidirectional brain–gut axis, enabling peripheral inflammatory stimuli, bacterial products or misfolded host proteins such as α-synuclein to reach the central nervous system and consequently promoting chronic systemic inflammation [107]. The resulting chronic smoldering systemic inflammation can promote blood–brain barrier permeability, thereby facilitating the infiltration of additional microbial metabolites and bacterial products into the cerebral parenchyma [108]. This mechanism is further compounded by the fact that bacterial amyloid proteins, such as curli, can directly enhance the accumulation of α-synuclein within both the enteric and central nervous systems, potentially accelerating neurodegenerative processes [109]. Consequently, the dysregulation of the gut–brain axis through these pathways may both initiate pathology and perpetuate a feedback loop where neuroinflammation further exacerbates sleep disruption and circadian misalignment [56,110]. The resulting chronic activation of microglia and astrocytes, stimulated by these peripheral insults, contributes to the persistent elevation of pro-inflammatory cytokines that can further compromise sleep architecture. The vicious cycle implies that therapeutic interventions targeting the modulation of gut microbiota and the restoration of intestinal barrier integrity may alleviate neuroinflammatory conditions and enhance sleep quality in patients with neurodegenerative diseases [111]. In addition, emerging reports indicate that truncal vagotomy could potentially lower the risk of Parkinson’s disease, showing the importance of this neural pathway in the development of synucleinopathies [94,112]. It should be emphasized that direct evidence for vagal propagation of misfolded protein pathology comes primarily from animal tracing and vagotomy studies, while human evidence remains largely indirect and epidemiological. Although neural transmission along the vagus nerve has received considerable attention, immune and metabolic signaling pathways also play critical roles in mediating communication between the gut and brain.

### 4.2. Neuroendocrine Signaling Along the Gut–Brain Axis

The resident gut microbiome can synthesize and regulate molecules such as serotonin [5-HT], γ-aminobutyric acid (GABA), and dopamine. These molecules may serve different functions in the gut than they do centrally. In the case of serotonin, approximately 90% of the body’s serotonin is produced by enterochromaffin cells in the gut, where it shapes motility, secretion, and mucosal homeostasis. In contrast, in the CNS, serotonin contributes to mood, pain, and sleep–wake regulation, and serves as the immediate precursor of melatonin [113]. While peripheral serotonin generally does not cross the blood–brain barrier, its significant peripheral role was previously downplayed but is now recognized [114]. The pineal gland is a known source of CNS melatonin, although extrapineal sources contribute most of the total melatonin produced in mammals [115]. Melatonin, synthesized peripherally in the gut, regulates gastrointestinal motility, protects mucosal cells from oxidative stress, modulates local immune activity, and exerts reciprocal effects on microbial composition [49,116]. Melatonin is highly lipophilic and can cross the blood–brain barrier and may even have a role in regulating the integrity of this barrier. Imbalances in GM can lead to dysregulation of these processes, as well as in the dysregulation of the HPA axis. On the other hand, the stress-induced glucocorticoid cascade and HPA misfiring will lead to inflammation, increased gut permeability and microbiome dysbiosis [117,118]. As discussed in Section 4.1, vagal signaling is an additional important regulator of these neuroendocrine interactions [119]. Orexinergic (hypocretin) signaling is another neuroendocrine interface between sleep–wake regulation and metabolic homeostasis, discussed further in the context of pharmacologic sleep interventions in Section 9.5.2. Originating in the hypothalamus and projecting to several different subcortical structures including the locus coeruleus, ventral tegmental area, dorsal raphe nuclei, tuberomammillary nucleus, basal forebrain, and ventrolateral preoptic nucleus, the orexinergic system is a key mediator of sleep–wake cycles, addiction, and motivation [120]. Two different orexin molecular subtypes exist, with the A and B forms both binding to and activating different postsynaptic G-protein-coupled receptors, Ox1R and Ox2R. This provides some variability in the possibility for drug modulation, where certain branches of the orexin network can be targeted more than others, thus possibly providing a desired therapeutic effect. Note that the neurons affected by this signaling utilize an array of different neurotransmitters themselves, including norepinephrine, dopamine, serotonin, histamine, acetylcholine, and GABA [121].

Ghrelin is a gastric-derived peptide hormone with central action on the hypothalamus but also extrahypothalamic sites. It is traditionally recognized for its appetite-stimulating property but also found to play a role in regulating circadian rhythm and sleep [122]. Ghrelin secretion follows a diurnal rhythm and has been associated with increased levels following sleep restriction in some studies [123]. Beyond its role in stimulating growth hormone secretion in humans, experimental administration of ghrelin has also been found to promote slow-wave sleep [124]. The gut microbiome can modulate enteroendocrine cells that produce ghrelin, and ghrelin in turn stimulates gut motility and epithelial barrier function, again supporting a bidirectional relationship [125]. In preclinical models of Parkinson’s disease, ghrelin has shown possible neuroprotective and anti-inflammatory properties, including preservation of dopaminergic neurons and reduced microglial activation [126,127], which, if verified in humans, could be an interesting therapeutic target [128].

### 4.3. Immune and Metabolic Communication Along the Gut–Brain Axis

The immune system is another major path through which alterations in gut microbial composition can influence sleep, brain function, and neurodegenerative processes. The inflammatory response itself, independent of direct protein transport, may influence cerebral function via the humoral pathway, where systemic cytokines and gut-derived inflammatory mediators exacerbate existing neurodegeneration. Specific microbial metabolites such as short-chain fatty acids—particularly butyrate, propionate, and acetate—are critical regulators of this axis. These SCFAs generally support beneficial effects by strengthening intestinal barrier integrity via tight-junction upregulation and exerting anti-inflammatory actions through HDAC inhibition and GPCR signaling; their reduced production during dysbiosis may therefore impair barrier maintenance and exacerbate systemic neuroinflammation [101,112,129,130]. Downstream effects, including on the blood–brain barrier and aquaporin 4 pathways, are detailed further in Section 6.3. The presence of curli-producing bacteria has also been shown to induce significant microgliosis and astroglial activation, suggesting that microbial amyloids actively accelerate the pathological burden in both the peripheral and central nervous systems [109,111,131,132].

The immune route is equally important: gut microbiota and their metabolites stimulate immune cells, including dendritic cells, T cells, and B cells, which modulate the host’s sleep patterns [133]. Gut microbiota diversity also correlates positively with both IL-6 levels and sleep duration [7]. The translocation of microbial products into systemic circulation can trigger the NLRP3 inflammasome, disrupting the intestinal barrier integrity and also helping the migration of immune cells to the central nervous system, where they exacerbate microglial activation and cognitive decline [130]. Under pathological conditions, these circulating proinflammatory molecules and immune cells cross the blood–brain barrier, triggering a chronic neuroinflammatory state that amplifies the activation of microglia and astrocytes [11]. This immune-mediated neuroinflammation is often compounded by the vagus nerve’s role in relaying peripheral inflammatory signals directly to the brainstem, which can independently trigger behavioral deficits and mood-related symptoms [134].

Gut microbiota influence host tryptophan metabolism and availability for conversion into serotonin, most of which occurs in the gut’s enterochromaffin cells. However, because peripheral serotonin does not appreciably cross the blood–brain barrier, this peripherally produced serotonin is not itself a direct precursor to CNS melatonin. CNS melatonin is synthesized locally, most readily by the pineal gland, from tryptophan and serotonin derived from local CNS metabolism rather than from the direct uptake of gut-derived serotonin [135]. Shifts in the microbial metabolism of tryptophan may influence CNS serotonergic and melatonergic pathways indirectly, an example of this being through the alteration of the peripheral tryptophan metabolite pool that may be used for CNS serotonin synthesis [136]. This happens via modulating flux through the kynurenine pathway and via immune and neural signaling mechanisms, rather than through direct entry of gut-derived serotonin into the brain [137,138,139,140]. In vitro, serotonin binding to 5-HT receptors on microglia can trigger the release of cytokine-carrying exosomes, providing a potential mechanism by which microbially influenced serotoninergic signaling could modulate neuroinflammation, although this is still to be established in vivo [141,142]. Microbial dysbiosis alters the kynurenine pathway flux, leading to the accumulation of neuroactive metabolites that exert pro-inflammatory effects and may impair synaptic plasticity [143]. Beyond these metabolic shifts, melatonin acts as a crucial regulator that can attenuate such neuroinflammatory states by reshaping intestinal microbiota and increasing the production of short-chain fatty acids. This cyclical interaction between melatonin and the gut microbiome suggests that therapeutic interventions aimed at stabilizing circadian rhythms could indirectly restore microbial homeostasis and dampen the associated inflammatory response [144,145]. Beyond neurotransmitters and short-chain fatty acids, additional microbially derived metabolites have emerged as important regulators of sleep and neurodegeneration.

### 4.4. Bile Acids and the Gut–Liver–Brain Axis

New evidence suggests that bile acids (BAs) also play an important role and may function as metabolic messengers within the gut–liver–brain axis, beyond their digestive role. A fraction of them can cross the blood–brain barrier and interact with receptors in the CNS, acting as an endocrine-like signaling modality. A healthy GM helps regulate this pool [146]. BAs also exhibit distinct circadian rhythmicity and have the capacity to influence CNS function by crossing the blood–brain barrier and by signaling via the VN pathways [147,148]. These metabolites, which are significantly shaped by gut microbial transformations, can modulate neurocircuitry governing arousal and sleep–wake regulation; for instance, BAs have been shown to promote wakefulness by blocking GABA_A_ receptors in the tuberomammillary nucleus of the hypothalamus [148]. Beyond that, aberrantly elevated bile acid levels in the CNS may disrupt the master circadian clock and promote neuroinflammation, contributing to the sleep fragmentation and metabolic dysregulation frequently observed in neurodegenerative pathologies [148,149,150]. Consequently, bile acid profiles are emerging as promising systemic biomarkers for the early detection of sleep disorders such as chronic insomnia and obstructive sleep apnea. Along with this, these profiles may serve as potential therapeutic targets for preserving cognitive resilience [148]. GERD leads to reflux of bile acids from the duodenum to the stomach and esophagus, particularly at night, creating disrupted sleep due to micro-arousals and local inflammation in the esophageal barrier. Sleep disruption and chronic insomnia in return will disrupt the GM and diversity of BAs, and all these combined factors are creating a vicious cycle [151,152,153,154]. As with other proposed gut–brain signaling mechanisms discussed in this review, the current evidence for bile-acid-mediated modulation of sleep and CNS function is derived mainly from preclinical models, with validation in human subjects remaining limited.

## 5. Microbial Contributions to Protein Aggregation

### 5.1. Bacterial Amyloids and Protein Cross-Seeding

Gut bacteria can produce amyloid, which functions as an antimicrobial peptide that plays a role in the local innate immune response [155,156]. Some enteric bacteria, but also some pathogenic bacteria, form a biofilm containing pathogenic amyloid proteins also known as curli, which serve as a defense mechanism for these bacteria [156,157,158,159]. In a healthy gut, they remain on the lumen side. Their production increases in case of stressors and can translocate to blood in a compromised gut environment [132]. Additionally, in mice models, gut amyloid levels have been shown to increase with age [160,161]. Bacterial amyloids differ from CNS amyloids in their primary structure but have some similarities in their tertiary structure as reported in the recent literature, which lead to the hypothesis of their participation in neurodegeneration. Increased gut permeability may lead to translocation of these proteins to the blood and subsequently into the brain. In preclinical models, this molecular mimicry has been proposed to allow bacterial amyloids to function as seed proteins that may exacerbate the misfolding of native amyloid proteins within the central nervous system, although direct evidence of this seeding mechanism in humans is still lacking [162,163]. This structural homology may also trigger cross-reactive immune responses and prime the immune system, whereby antibodies generated against bacterial amyloids inadvertently target endogenous amyloid-β peptides, accelerating neuroinflammatory cascades [131,164,165]. This priming effect on the immune system may lead to an enhanced response to CNS amyloid and microglia dysregulation [159,166]. Beyond structural cross-reactivity, the local increased permeability, as well as the loss of protective commensals, increase the systemic translocation of bacterial endotoxins like LPS, which eventually cross the blood–brain barrier. This suggests a potential LPS-induced neuroinflammation, with bacterial LPS increasing the CNS LPS, particularly in the neocortex and hippocampus [99,167,168]. The loss of some protective commensals, with a potential dampening effect on LPS via the inhibition of the histone deacetylase (HDAC) pathway, may also contribute to the inflammation cycle [167]. Such systemic immune activation is further compounded by the observation that coincubation of Aβ with bacterial LPS potentiates fibril formation, directly helping the progression of amyloidosis [169,170]. This synergy between microbial endotoxins and amyloidogenic proteins may explain why many individuals harbor significant cerebral amyloid deposits without exhibiting overt cognitive decline, as the clinical phenotype often depends on the subsequent microglial inflammatory response [131].

Bacterial-derived amyloid may also trigger increased aggregation of synuclein both in the gut and in the brain [171]. Given the intimate link between enteric dysbiosis and neuroinflammation, the systemic release of bacterial endotoxins such as LPS can promote dopaminergic neuronal death by activating inflammatory pathways and microglia [172]. LPS is a critical mediator that triggers alpha-synuclein aggregation within the enteric nervous system, effectively bridging systemic inflammation and pathological protein accumulation [103,173]. In addition to promoting protein aggregation, microbial products may influence neurodegeneration through direct neural communication pathways linking the gut and the brain.

Microbial products may influence neurodegeneration through multiple mechanisms, including immune priming, protein cross-seeding, blood–brain barrier disruption, and neuroinflammation. Although most attention has focused on the gastrointestinal microbiome, emerging evidence suggests that microbial communities at other barrier surfaces may also contribute to neurodegenerative disease risk. Most of this evidence derives from cell-based and animal models, and the extent to which bacterial amyloid cross-seeding contributes to human neurodegenerative disease remains to be established.

### 5.2. Oral Microbiome, Periodontal Disease, and the Oral–Brain Axis

Although most research has focused on the GI microbiome, other barrier-associated microbial communities may similarly influence neurodegeneration. Beyond the gut, the oral microbiome may also play a secondary role in this pathophysiology via the oral–brain axis, disseminating microbial products directly into systemic circulation or through the trigeminal and vagus nerves. Initial periodontitis activates the host immune response and leads to modification of the subgingival microbiota, which in turn causes subsequent chronic inflammation. As in the gut, microbial invasion leads to a disrupted epithelial barrier and blood translocation of pathogens and inflammatory agents to the CNS, or via direct trigeminal and vagus nerve pathways [174,175]. This neurological access facilitates the colonization of the CNS by pathogens such as *Porphyromonas gingivalis* among others, which can induce profound neuroinflammatory shifts and exacerbate the amyloidogenic processes and tau hyperphosphorylation associated with cognitive impairment [176,177,178,179]. Porphyromonas gingivalis has been shown to disturb day/night microglial activity, providing a mechanistic link between chronic oral infection and the disruption of glymphatic cleansing systems essential for amyloid clearance [180]. *P. gingivalis* affects microglial phagocytic activity by impairing the nocturnal efficiency of the glymphatic system, which is critical for the clearance of interstitial solutes and neurotoxic protein aggregates [180]. This impairment potentially exacerbates the pathogenesis of neurodegenerative conditions, as chronic oral dysbiosis may promote the accumulation of misfolded proteins and axonal damage through systemic inflammatory signaling [181,182]. *P. gingivalis* causes direct neuroinflammatory processes that promote the synthesis of pro-inflammatory cytokines, further compromising blood–brain barrier integrity and facilitating the infiltration of additional systemic immune cells into the cerebral parenchyma [183]. This led to reactive gliosis and chronic neuroinflammation, potentially aggravating the accumulation of amyloid-beta and the subsequent development of AD-type pathologies [184,185]. Reactive astrogliosis induced by *P. gingivalis* may potentially lead to depolarization and loss of aquaporin-4 polarization at the astrocytic end-feet, which directly undermines the convective flow required for efficient glymphatic clearance [186].

Peripheral periodontal infections can then prime microglial cells, which enhances their reactivity to subsequent systemic inflammatory insults and accelerates neurodegenerative progression [187,188]. Li et al. showed some association between OSA and salivary microbiota, though direct causal relationships will need further longitudinal evaluation [189]. This had been already previously reported by Ko et al. in 2022, particularly with elevated *Porphyromonas* and *Aggregatibacter*, as well as proinflammatory cytokines [190]. These findings can also be integrated in a broader conceptual framework, including small vessel disease as chronic inflammation, and *Porphyromonas gingivalis* have been found associated with increased atherosclerotic diseases and cardiovascular diseases, reinforcing the systemic disease paradigm over an organ-only approach [179,191].

## 6. Role of the Glymphatic System

### 6.1. Glymphatic Physiology and Sleep

The glymphatic system is a highly organized fluid transport network in which cerebrospinal fluid (CSF) enters the brain parenchyma along arterial perivascular spaces, mixes with interstitial fluid (ISF), and exits via perivenous pathways. This serves to carry metabolic waste to the periphery and cervical lymph nodes [94]. This process is highly dependent on aquaporin-4 (AQP4) channels concentrated on astrocytic endfeet, facilitating rapid CSF–ISF exchange. Without these channels, clearance of amyloid-β (Aβ) in AQP4 knockout mice has been shown to be significantly impaired, which led to a possible path of interest when considering the current models of how amyloid-related pathologies, such as Alzheimer’s, develop [192,193]. This system works in conjunction with meningeal lymphatic vessels, which drain CSF components and waste proteins into the cervical lymph nodes, serving as the final conduit out of the CNS [94,194]. Consequently, dysfunction at any point along the glymphatic pathway may promote the accumulation of toxic proteins, inflammatory mediators, and metabolic waste products that contribute to neurodegeneration.

Sleep is perhaps one of the most influential moderators of the glymphatic system. In mice studies, glymphatic flow is reported to be minimal during awake time and increasing significantly during non-rapid eye movement (nREM) sleep, which allows the interstitial extracellular fluid spaces to expand and CSF–interstitial space exchange to occur. This is driven by a reduction in norepinephrine (NE) tone [195]. Slow wave activity has a positive correlation with glymphatic influx, while elevated NE during wakefulness actively suppresses this influx [196]. During SWA, the interstitial space expands by up to 60% of its original volume, which optimizes conditions for waste clearance, including the removal of Aβ and tau [62]. Conversely, a single night of sleep deprivation measurably increases brain Aβ burden in humans [197,198]. Disruption of the slow waves, as well as of the slow vasomotion (the rhythmic contraction and dilation of the arterial wall), will affect waste clearance and raise CSF Aβ levels [199]. More recent human studies have produced divergent findings, indicating that the magnitude and consistency of this sleep–wake difference may be more variable than initially suspected.

### 6.2. Glymphatic Dysfunction in Neurodegenerative Disease

**Figure 3:**
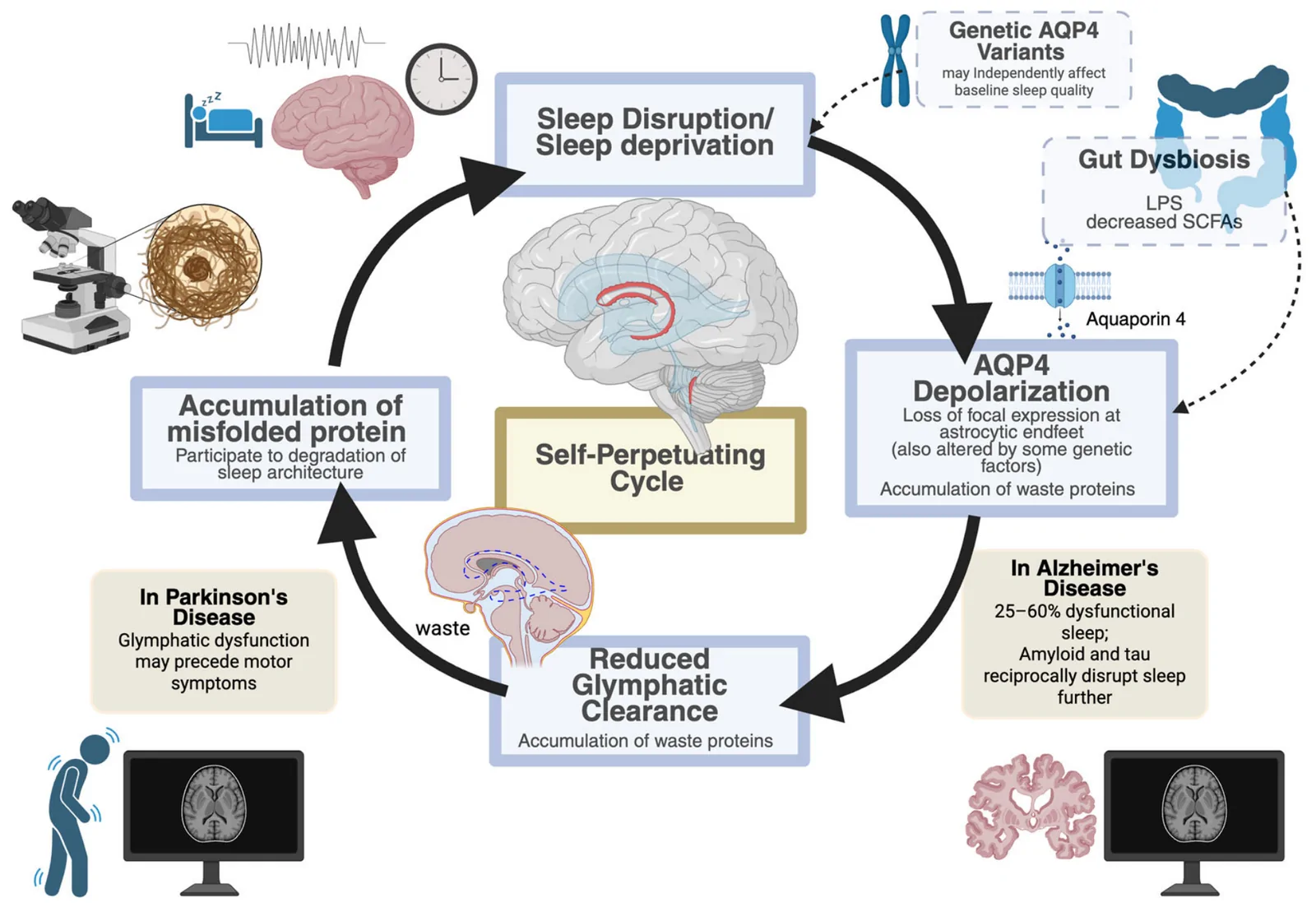
Glymphatic dysfunction vicious cycle, where sleep disruption promotes AQP4 depolarization, reducing glymphatic clearance and causing waste buildup, which in turn further perpetuates sleep degradation, modulated by AQP4 genetic variants and gut dysbiosis.

The relationship between sleep, glymphatic dysfunction, and neurodegeneration is bidirectional and self-perpetuating (Figure 3). Between 25 and 60% of AD patients present with dysfunctional sleep. Reciprocally, Aβ and tau accumulation further disrupts sleep architecture, establishing a feed-forward cycle in which impaired glymphatic clearance accelerates amyloid deposition, which in turn further degrades sleep quality and glymphatic function [195,196,200,201,202]. Additionally, AQP4 depolarization appears to be a key contributor in this loop. Genetic variants in AQP4 alter sleep quality, and extended sleep deprivation over 48 h causes AQP4 depolarization and subsequent loss of its localized expression at astrocytic endfeet, which in turn reduces glymphatic clearance and promotes Aβ accumulation [195,196]. Similarly, in PD, glymphatic dysfunction appears to possibly precede motor symptoms, and α-synuclein itself inhibits glymphatic flow, creating an analogous vicious cycle of clearance failure and toxic protein accumulation [62,203,204,205,206]. This temporal pattern is consistent with body-first models of synucleinopathy discussed in Section 3, in which peripheral and glymphatic dysfunction may precede central pathology.

### 6.3. Microbiome Regulation of Glymphatic Function

Recent evidence suggests that the gut microbiome may constitute an important upstream regulator of glymphatic function. A healthy microbiome produces short-chain fatty acids (SCFAs) such as butyrate, acetate and propionate, which are thought to protect the blood–brain barrier and additionally act to maintain waste clearance from the brain by preserving the polarization of AQP4 channels [29,62]. The polarized localization of AQP4 channels at the astrocytic endfeet that envelop the cerebral microvessels is essential to proper exchange between the cerebrospinal fluid (CSF) and the interstitial space, regulating the clearance of metabolic waste like amyloid and other proteins. Inflammation and other pathological processes will impair the glymphatic flow leading to an accumulation of these waste proteins. SCFAs, via the aryl hydrocarbon receptor (AhR) activation pathway and enhancement of tryptophan-derived AhR ligands at astrocytes level support the integrity of the system [26]. SCFAs also help in regulating the tight-junction proteins and strengthening the blood–brain and blood–CSF barriers. Conversely, gut dysbiosis elevates lipopolysaccharides (LPSs) and pro-inflammatory cytokines (IL-6, TNF-α) that manage to cross the blood–brain barrier and lead to neuroinflammation and subsequent accumulation of neurotoxic proteins in the CNS [163]. In animal models, a leaky barrier allowing infiltration of these pro-inflammatory cytokines has been shown to trigger reactive astrogliosis and AQP4 mislocalization. SCFAs, by tightening these barriers, contribute to the maintenance of vascular astrocytic endfeet integrity, which helps in preventing the AQP4 depolarization that often accompanies chronic neuroinflammation [207]. Reactive astrogliosis compromises glymphatic drainage through the downregulation of polarized AQP4 expression. By preserving homeostatic SCFA levels, one can promote microglia to remain in a neuroprotective state rather than transitioning into this deleterious inflammatory phenotype [208,209]. Largely, evidence for microbiome-mediated modulation of glymphatic function and AQP4 polarization stems from preclinical rodent studies. In this case, direct evidence of linkage between microbiome composition and glymphatic clearance in humans remains limited. The interactions among sleep, the microbiome, and glymphatic function are further shaped by biological and environmental factors that influence disease susceptibility across the lifespan.

## 7. Biological Modifiers of the Gut–Brain–Sleep Axis

### 7.1. Aging and Age-Related Dysbiosis

Multiple physiological and environmental states predispose individuals to gut dysbiosis and impaired neuroprotection. Aging is one of the most important considerations, as the gut microbiome evolves across the lifespan and becomes increasingly individualized, with shifts being influenced by immune function, metabolism, and disease states [30]. Dysbiosis has been associated with systemic diseases such as diabetes and inflammatory bowel disease, which are both linked to chronic inflammation and an increased risk of neurodegenerative disorders [30,77]. These inflammatory conditions may impair neuronal resilience through cytokine signaling, oxidative stress, and disruption of gut–brain communication pathways. Specifically, microbial metabolites, including tryptophan derivatives and bile acids, have been shown to modulate microglial activation and astrocytic support systems, providing mechanistic links between systemic metabolic shifts and central nervous system integrity [147]. While aging is a major determinant of microbiome composition, biological sex serves as another important source of variability that influences gut–brain communication throughout the lifespan.

### 7.2. Sex Differences in the Gut Microbiome

The gut microbiome exhibits a sexual dimorphism that emerges after puberty and is strongly influenced by sex hormones [31]. Estrogen, progesterone, and androgens interact bidirectionally with the microbiome: hormones shape microbial composition, while microbial enzymes (e.g., β-glucuronidases within the estrobolome) regulate circulating hormone levels through enterohepatic recirculation [31]. The estrobolome, a concept first coined in 2011 by Plottel and Blaser then later Kwa et al. in 2016 [210,211], refers to the specific kind of gut microbes responsible for metabolizing and modulating systemic estrogen levels [32,33]. This microbial network acts as a primary regulator in the gut–liver–brain axis and via the estrogen’s enterohepatic recirculation, determining the bioavailability of active estrogens in the bloodstream [212,213,214]. These interactions influence immune signaling, metabolism, and neuroendocrine pathways, contributing to sex-specific susceptibility to both gastrointestinal and neurological disease.

### 7.3. The Estrobolome and Estrogen Metabolism

The estrobolome is gaining more attention, as mentioned above, and particularly in context of sex-related differences seen in some neurodegenerative processes, like AD. The central function of the estrobolome is mediated by the bacterial enzyme beta-glucuronidase [32,213]. The liver filters estrogen from the blood and deactivates them by glucuronidation. It is then excreted via the bile into the small intestine. Once in the gut, the estrobolome removes the glucuronic acid from the estrogens via beta-deconjugation, turning it back into free active estrogen that can cross the intestinal lining and pass back into the bloodstream [213,215]. Gut dysbiosis can alter beta-glucuronidase activity; specifically, a reduction in activity can lead to hypoestrogenic states [32], which are associated with metabolic syndrome [216,217] and potentially with brain fog and cognitive decline [218,219]. Conversely, hyper-activity of these bacterial enzymes may contribute to estrogen-dependent pathologies including cancer, highlighting the delicate balance required for hormonal homeostasis [211,220]. This led to the bidirectional pathway denomination of the estrobolome–brain axis, illustrating how these microbial communities functionally bridge the endocrine system and the central nervous system [221]. It is considered as a potential influencer in pathophysiology of sex-specific neurodegenerative disorders, as hormonal modulation of the gut microbiome directly impacts neuroimmune pathways and microglial functionality [222]. In Alzheimer’s disease, differences in microbiomes between gender may be a significant contributor for the higher prevalence among women [214]. During menopause, the decline in estrogen disrupts the gut–estrogen–brain axis, possibly explaining mood shift, brain fog and cognitive symptoms, as well as systemic inflammation [223,224]. Preclinical research indicates that the depletion of estrogen signaling leads to alterations in the microglial phenotype, specifically diminishing the presence of plaque-associated microglia, which is paralleled by a reduction in neurodegenerative pathology [225]. Understanding these interactions is leading to new research into microbiome-based interventions, such as targeted probiotics, to support brain health during hormonal transitions [224].

### 7.4. Menopause as a Vulnerable Transition

Menopause is a particularly vulnerable state for gut–brain axis disruption. Declining estrogen and progesterone levels, along with increased gonadotropins such as follicle-stimulating hormone (FSH), are associated with reduced microbial diversity, shifts in microbiome composition, and increased similarity to male microbial profiles [31]. Estrogen normally exerts protective effects on gastrointestinal function, including regulation of motility, mucosal integrity, immune responses, and microbial balance; its loss contributes to increased intestinal permeability, immune activation, and dysbiosis [226]. Clinically, menopausal women exhibit higher rates of functional gastrointestinal disorders and gut–brain interaction disorders, with symptoms such as visceral hypersensitivity, altered motility, and increased inflammation [227]. Additionally, hormonal changes during menopause contribute directly to neurodegenerative risk. Elevated FSH levels have been implicated in promoting neuroinflammation, lipid dysregulation, and amyloid pathology, which may lead to an increased susceptibility to Alzheimer’s disease [228,229]. Elevated FSH and drops in estrogen levels are associated with an increased gut permeability and dysbiosis [220], which may compound the neuroinflammatory and amyloidogenic mechanisms described above, particularly in women. Sleep disruption, which is more prevalent during menopause, further compounds this risk, as it is associated with impaired glymphatic clearance of neurotoxic proteins and increased Alzheimer’s pathology [230]. Mechanistically, this estrogen-dependent increase in gut permeability and immune activation is proposed to promote the same systemic inflammatory and microglial changes described in Section 5 and Section 6 of this review. This includes LPS-driven neuroinflammation and altered microglial phenotypes, which are linked to amyloid deposition and glymphatic impairment. This provides a plausible, though not yet validated, link between estrobolome dysregulation and AD-related pathology in women.

### 7.5. Environmental and Lifestyle Influences

Environmental and lifestyle exposures further exacerbate GBA dysfunction. Environmental pollutants such as heavy metals, endocrine disruptors, and particulate matter can directly alter microbiome composition, increase oxidative stress, and disrupt intestinal barrier integrity, leading to systemic inflammation and neurotoxicity [34,35]. These exposures may also impair communication along neural, endocrine (e.g., hypothalamic–pituitary–adrenal axis), and immune pathways, contributing to psychiatric disorders and cognitive decline. Early-life exposures are also particularly impactful, as microbiome development during infancy plays a key role in immune and neuroendocrine programming [77,231].

## 8. Emerging Biomarkers of the Gut–Brain–Sleep Axis

Given the complexity of the microbiota–gut–brain–sleep axis, no single biomarker is likely to adequately capture disease risk or progression. Instead, emerging evidence supports the use of multidimensional biomarker panels integrating microbial composition, microbial metabolites, intestinal barrier integrity, hormonal regulation, sleep physiology, autonomic function, and neuroimaging measures, as further detailed below (Table 2). These biomarkers differ substantially in their stage of validation and current clinical use: some (e.g., fecal calprotectin) are already used clinically for other gastrointestinal indications, others (e.g., DTI-ALPS, bile acid profiling) are active research tools not yet validated for clinical decision-making in neurodegenerative disease, and still others (e.g., beta-glucuronidase activity, the “Alzheimer’s dysbiosis index”) remain candidate mechanistic markers proposed largely on a theoretical basis.

### 8.1. Microbiome and Metabolite Biomarkers

Identifying molecular signatures of dysbiosis, such as altered ratios of short-chain-fatty-acid-producing bacteria and elevated levels of circulating lipopolysaccharides, offers a window into the systemic inflammation driving central nervous system pathology [33]. Bile acid profiling on serum and stool samples using liquid chromatography–mass spectrometry, allows for the quantification of secondary bile acid derivatives, which serve as direct readouts of the gut-microbiota metabolic capacity [232,233,234]. Bile acid profiling also serves as a non-invasive diagnostic tool to evaluate sleep pathologies like sleep apnea, chronic insomnia, and altered pattern in the circadian clock [148]. While microbial composition and metabolite profiles provide information about microbiome function, biomarkers of intestinal barrier integrity may offer insight into one of the key mechanisms linking dysbiosis to systemic inflammation.

### 8.2. Intestinal Barrier Biomarkers

The quantification of fecal calprotectin and zonulin levels serve to delineate the degree of intestinal permeability, providing a functional assessment of the barrier integrity that often precedes clinical neurocognitive manifestations [235,236]. These findings suggest that specific microbial signatures might be used as potential biomarkers for the early detection of cognitive decline, particularly as these disturbances often precede the clinical manifestation of neurodegenerative symptoms [47]. Given the growing recognition of sex-specific influences on neurodegenerative disease risk, biomarkers reflecting hormone–microbiome interactions have attracted increasing attention.

### 8.3. Sex Biomarkers

Physiological and sex-specific differences complicate the utility of these biomarkers, as hormonal profiles likely dictate the threshold at which microbial metabolites trigger neuroinflammatory cascades [236]. Consequently, personalized therapeutic strategies should integrate the understanding of hormonal fluctuations and microbiome dynamics into treatment plans to optimize patient outcomes [225]. Longitudinal monitoring of the estrobolome, specifically the functional expression of microbial beta-glucuronidase measured using spectrophotometric or fluorometric assays on a stool sample, may offer a non-invasive index for assessing systemic estrogen availability and the associated neuroprotective status in menopausal women, though this remains a candidate mechanistic marker rather than a clinically confirmed one [237]. High enzyme activity indicates a greater capacity for estrogen reabsorption, which may lead to estrogen dominance or higher circulating estrogen levels. Genetic profiling or metagenomics, and DNA sequencing of the GM streamline a comprehensive evaluation of the microbial genes encoding β-glucuronidase, enabling a more precise assessment of how gut-derived metabolites influence the systemic bioavailability of hormones [212].

### 8.4. Imaging Biomarkers of Glymphatic Function and Neuroinflammation

Imaging biomarkers including magnetic resonance imaging (MRI)-based diffusion tensor imaging analysis along the perivascular space (DTI-ALPS) index, initially reported by Taoka et al. in 2016 [238], as well as positron emission tomography targeting translocator protein (TSPO-PET) [239,240,241], are gaining momentum in assessing glymphatic system dysfunction and neuroinflammation [238,242,243,244,245]. These advanced diagnostic modalities can facilitate the early detection of increased blood–brain barrier permeability, which is linked with sleep and also thought to be a critical precursor to hippocampal atrophy and cognitive dysfunction. Gao et al. showed that DTI-ALPS indexes were impaired in patients with insomnia compared to healthy controls, supporting the use of this technique, despite some limitations and fine tuning [246]. In the same study, they used the same imaging technique to follow response to treatment with repetitive transcranial magnetic stimulation (rTMS), suggesting a potential promising role in both its diagnostic and mechanistic value, as well as in monitoring responses to therapeutic interventions. Recent work by Jin et al. using DTI-ALPS demonstrated glymphatic dysfunction and cognitive decline associated with chronic insomnia in middle-aged and older adult cohorts [247]. The study by Xiong et al. supports using the average ALPS index as an indirect indicator associated with glymphatic dysfunction in insomnia, potentially aiding prediction and stratification in cohorts, although it does not directly quantify glymphatic flow [248]. These imaging advancements may allow researchers to correlate glymphatic clearance rates with specific hormonal profiles, potentially uncovering how menopausal transitions modulate interstitial fluid exchange and subsequent protein aggregation [249,250].

By characterizing these pathways, clinicians may leverage the MGB axis as a therapeutic target, potentially mitigating systemic inflammation through targeted modulation of the gut microbiota [251,252]. Specifically, the development of an “Alzheimer’s dysbiosis index”—a composite score based on the ratio of specific bacterial taxa—could refine the prognostic accuracy of identifying individuals at risk for rapid cognitive decline [147], although this index is currently a theoretical construct rather than a validated clinical tool. In this context, analyzing specific microbial species such as *Clostridium leptum* and *Lachnospiraceae* may provide high-resolution predictive data for amyloid-beta and tau-related pathological burden [253]. Similarly, the assessment of intestinal permeability through tight junction protein expression patterns can be used as a diagnostic indicator of both blood–brain and gut barrier integrity in AD [208]. While longitudinal studies are essential to determine whether these microbiota–gut–brain axis alterations are upstream or downstream of pathological brain changes, incorporating fecal calprotectin levels may enhance the clinical assessment of intestinal permeability as a precursor to systemic neuroinflammation [254].

### 8.5. Sleep and Autonomic Biomarkers

Sleep, autonomic regulation, and vagal signaling biomarkers may provide predictive value. Sleep biomarkers could be integrated utilizing polysomnography to correlate fragmented sleep architectures with fluctuations in gut-derived metabolites and systemic inflammatory markers [255]. Combining multi-omics indicators with established diagnostic criteria has been proposed as a way to validate findings across diverse patient populations [256]. However, the existing human studies disagree on which bacteria phyla are altered, possibly due to the small-sample-size studies and inconsistent methods used across them. Larger, high-powered, multi-region studies are needed to clarify specific details before this approach can be considered diagnostically reliable [257].

Vagal nerve imaging has emerged as a promising technique for evaluating the structural and functional integrity of the parasympathetic pathway, which can act as a path for microbial signaling to the brain [258]. Advanced neuroimaging techniques may clarify how age-related changes in vagal tone correlate with the systemic inflammatory profiles observed in patients with varying degrees of cognitive impairment [259]. Addressing these structural variations is vital, as the functionality of the gut–brain axis depends heavily on the precise orchestration of these neural circuits [260]. Complementary to vagal nerve imaging, some data on electrogastrography (EGG) suggest that it could be used as a tool in early detection of prodromal or early-stage untreated PD, as these patients show dysmotility and irregular gastric slow waves [261,262]. Slow waves are mediated by the vagal nerve, and EGG is another noninvasive way to assess its activities [261].

Vagal nerve and sleep imaging markers could help elucidate the temporal relationship between autonomic nervous system dysfunction and the onset of systemic inflammatory signaling. Well-designed longitudinal investigations employing these comprehensive approaches are necessary to establish clear directionality and causality between gut microbial community structure and neurological outcomes [263]. To refine these diagnostic models, future research must incorporate clinically well-characterized cohorts to address the confounding effects of diet, sex, and disease severity [264]. The integration of state-of-the-art meta-omics, including metagenomics and metabolomics, will be essential to map the complex microbial signaling pathways that mediate host–environment interactions [265,266]. Ultimately, no single biomarker is likely to fully capture the complexity of the microbiota–gut–brain–sleep axis. Future diagnostic approaches will likely integrate microbiome composition, microbial metabolites, intestinal permeability markers, hormonal profiles, sleep metrics, autonomic measures, and neuroimaging data into multidimensional biomarker panels. Many of the pathways presented in these biomarker panels are also emerging therapeutic targets, raising the possibility of biomarker-guided intervention strategies.

## 9. Therapeutic Opportunities Along the Gut–Brain–Sleep Axis

The interventions discussed in this section vary considerably in their stage of clinical translation. Lifestyle interventions (Section 9.6) and clinically approved sleep pharmacotherapies, such as dual orexin receptor antagonists, melatonin receptor agonists, and low-dose doxepin (Section 9.5), are already used clinically, although their disease-modifying effects on the gut–brain–sleep axis remain to be formally established. Vagus nerve stimulation, particularly its non-invasive transcutaneous form, has been studied clinically for other indications and is being explored experimentally for gut–brain axis modulation. In contrast, fecal microbiota transplantation, probiotic and stem-cell-based microbiome therapies, estrobolome-targeted interventions, bile acid modulation, and glymphatic enhancement strategies, such as AQP4 modulation, hyperosmolar therapy, and meningeal lymphatic and photobiomodulation approaches, remain largely preclinical or at an early experimental stage. Thus, limited or no controlled human trial data exists for these treatments within neurodegenerative disease populations. This variability of evidence should be considered when interpreting the translational potential of each approach discussed below. Future stratification of patients by brain-first versus body-first phenotype (Section 3) may eventually help match individuals to gut-directed versus centrally directed interventions, although this approach has not yet been tested prospectively.

### 9.1. Microbiome-Based Therapies

#### 9.1.1. Fecal Microbiota Transplantation

Beginning in the gut itself, fecal microbiota transplantation (FMT) is one of the most direct approaches to modulating the gut microbiome. This intervention is still considered experimental in the context of neurodegenerative disease. As mentioned earlier, preclinical studies transplanting healthy donor microbiota into animal models have demonstrated improvements in motor function, increased striatal neurotransmitter concentrations, and reduced inflammatory markers [267]. In autism spectrum disorder (ASD), open-label clinical trials have shown sustained improvements in both gastrointestinal and behavioral symptoms lasting up to two years post-treatment [267,268]. While not a neurodegenerative disorder, ASD is associated with cognitive challenges, thus possibly serving as a good model for fluctuations in this cognition. However, the absence of large-scale, double-blind randomized controlled trials for most neurodegenerative disorders substantially limits the generalizability of current findings, and concerns regarding donor selection, procedural safety, and long-term microbiome engraftment remain unresolved [267].

#### 9.1.2. Probiotics and Prebiotics

Probiotic and prebiotic interventions offer a potentially more accessible alternative, though evidence in humans remains more elusive, with most of the mechanistic data coming from mouse models. Proposed mechanisms include enrichment of the local microbiota by bacteria such as lactobacilli, which indirectly produce potentially protective byproducts like butyrate; reduction of bacteria with potentially harmful byproducts; and an increase in SCFA-producing bacteria like *Blautia faecicola*. These shifts have been associated with attenuated neuroinflammation cascades in both AD and PD [269,270,271,272,273]. While evidence from animal models is encouraging, clinical results in patients with neurodegenerative disorders such as AD or PD remain inconsistent, reflecting variability in strain selection, dosing, and patient-specific microbiome composition, as well as reflecting the need for a standardized protocol that is still tailored to the host [266,274,275,276,277]. While probiotics and prebiotics are already widely available and used clinically for other gastrointestinal indications, they are not yet clinically validated by proper clinical trials as disease-modifying therapies for neurodegenerative disease.

#### 9.1.3. Microbiome-Targeted Stem Cell and Strain-Specific Therapies

Stem cell therapy is a particularly innovative GBA-targeted strategy. Intravenously administered stem cells in PD models have demonstrated preferential migration to gut tissue, where they reduced inflammatory microbiota and dampened inflammation in both the gut and brain simultaneously [269]. In AD models, administration of specific Bifidobacterium strains has been shown to reduce amyloidosis, enhance synaptic plasticity, and improve cognitive function [269]. Despite these promising findings, challenges related to cell sourcing, dosing optimization, and potential tumorigenic risk continue to hinder clinical translation.

While microbiome-based interventions target upstream drivers of gut–brain dysfunction, additional strategies aim to restore endocrine and circadian regulation.

### 9.2. Endocrine and Metabolic Therapies

#### 9.2.1. Melatonin Supplementation

Exogenous melatonin supplementation has also been explored as a neuroprotective adjunct. Both exogeneous melatonin and its synthetic counterpart act primarily on MT1 and MT2 melatonin receptors densely expressed in the suprachiasmatic nucleus of the hypothalamus and mediating their shared chronobiotic, with established roles in circadian rhythm regulation, and sleep-promoting effects. Melatonin has shown some efficacy in reducing sleep onset latency and increasing total sleep time in secondary sleep disorders, which support the sleep-dependent processes critical to brain waste clearance and neurological homeostasis [278]. Its effects on sleep efficiency, however, remain negligible, and optimal dosing for aging populations is still unclear [249,278]. While not disease-modifying, exogenous melatonin use can be viewed as a supplement to encourage brain health via the sleep–brain axis.

#### 9.2.2. Estrobolome-Targeted Therapies

Growing recognition of the estrobolome has stimulated interest in interventions aimed at preserving gut–estrogen signaling. Estrobolome regulation in menopause using selective estrogen receptor modulators or targeted probiotics may address the decline in systemic estrogen levels that otherwise impairs metabolic homeostasis and contributes to neurodegenerative risk factors [279,280]. This, however, remains just an interesting proposal. At this time, no clinical trials have been conducted to explore this treatment modality.

#### 9.2.3. Bile Acid Modulation

Bile-acid-targeted interventions, which remain at a preclinical stage of development, could in principle act on gut–brain–liver axis by modulating metabolic signals that cross the blood–brain barrier to influence hypothalamic centers responsible for systemic energy balance and neuroendocrine regulation [12]. The modulation of bile acid signaling pathways can influence hepatic-derived factors that mitigate gut dysbiosis and then reduce systemic inflammation that might otherwise compromise glymphatic efficacy [281]. In addition, new findings have shown that bile acids can influence sleep and arousal by crossing the BBB and modulating neurocircuitry governing arousal, contributing to sleep fragmentation [148]. This mechanism sheds a light on the necessity of precise temporal regulation of bile acid pathways to avoid disrupting the homeostatic synchronization between metabolic activity and restorative sleep cycles [148].

Beyond endocrine pathways and building on the glymphatic physiology and microbiome–glymphatic interactions discussed in Section 6, increasing attention has focused on therapeutic strategies that directly target glymphatic clearance mechanisms.

### 9.3. Glymphatic Enhancement Strategies

#### 9.3.1. Aquaporin-4 Modulation

When considering glymphatic modulation, aquaporin-4 (AQP4) exemplifies a prominent avenue of investigation. The AQP4 facilitator TGN-073 has demonstrated the capacity to promote interstitial fluid circulation within the blood–brain barrier, while the AQP4 inhibitor TGN-020 has been shown to reduce ischemic cerebral edema and infarct volume in rodent stroke models, an application directed at acute edema control by limiting astrocytic water influx rather than augmenting perivascular clearance [282,283]. A major challenge, however, is that AQP4 is expressed throughout peripheral tissues, including skeletal muscle and renal epithelium, increasing the risk of off-target effects and complicating the development of CNS-specific pharmacological agents [282,283].

#### 9.3.2. Hyperosmolar Therapy

Systemic hyperosmolar therapy using hypertonic saline may allow for enhanced glymphatic drug delivery. This therapy has been shown to produce approximately a three-fold increase in agent influx and a five-fold increase in anti-amyloid antibody binding in preclinical models [282]. While inexpensive and non-complicated, the risk of CNS demyelination limits safety.

#### 9.3.3. Meningeal Lymphatic and Photobiomodulation Approaches

Enhancement of meningeal lymphatic vessel (MLV) function via vascular endothelial growth factor C (VEGF-C) has shown efficacy in aged murine models, improving CSF drainage, cognitive performance, and amyloid-β clearance [284]. Photobiomodulation therapy targeting MLVs with near-infrared light has similarly reduced amyloid deposition and improved cognitive outcomes in Alzheimer’s disease mouse models. However, challenges related to cranial scattering and wavelength optimization remain unresolved [284]. Physical therapies, including repetitive transcranial magnetic stimulation, have also shown promise in enhancing glymphatic excretory efficiency in preclinical settings. Although AQP4 modulation, hyperosmolar therapy, photobiomodulation and VEGF-C have shown some preclinical evidence of efficacy, none have been tested yet in human clinical trials.

### 9.4. Neuromodulation of the Gut–Brain Axis: Vagal Nerve Stimulation

As introduced in Section 4.1, the vagus nerve plays a key role in the bidirectional gut–brain communication; this section focuses on its therapeutic modulation. Vagal nerve electrical stimulation has shown some potential in augmenting global glymphatic influx, with particularly pronounced effects observed in the rostral brain regions [285]. Vagal nerve neurostimulation may further influence cerebrovascular pulses and AQP4 polarization, potentially helping enhanced cerebrospinal fluid–interstitial fluid exchange, similar to high-frequency audiovisual stimulation. The release of vasoactive intestinal peptide and the activation of adenosine A2AR receptors by such neural engagement have been identified as key mediators in sustaining these neurovascular responses [286,287]. Beyond invasive methodologies, transcutaneous vagal nerve stimulation (tVNS) has emerged as a promising non-invasive alternative, demonstrating efficacy in modulating autonomic balance, mitigating systemic inflammation through the cholinergic anti-inflammatory pathway, and strengthening intestinal barrier integrity, which supports the gut–brain–liver axis [288]. In addition, research suggests that tVNS can beneficially influence sleep architecture, potentially by reducing nocturnal awakenings and increasing sleep efficiency. This indirectly facilitates the glymphatic clearance processes occurring during deep sleep [289,290]. By combining these targeted neuromodulation techniques with lifestyle approaches that leverage vagal tone—such as slow-paced breathing or targeted physical interventions—researchers are exploring synergistic strategies to stabilize systemic homeostasis, improve circadian rhythmicity, and ultimately offer neuroprotective benefits in aging populations [288,291]. Non-pharmacological modalities such as exercise and the strategic regulation of polyunsaturated fatty acid intake have also been shown to modulate cerebrospinal fluid–interstitial fluid exchange, showing the importance of a multi-faceted approach to glymphatic enhancement [283].

### 9.5. Sleep-Focused Therapies

Because sleep is a central regulator of glymphatic clearance, neuroinflammation, and gut microbial homeostasis, pharmacologic treatment of sleep disturbances is an important but often underappreciated therapeutic strategy. However, not all hypnotic agents exert equivalent effects on the interconnected pathways discussed throughout this review. Table 3 details some of the effects of specific hypnotic agents among other interventions. It should be emphasized that the evidence underlying Table 3 varies substantially in quality and maturity across agents and outcome measures. This ranges from mechanistic rodent data to limited human biomarker studies. Therefore, the table should be interpreted as a qualitative synthesis of current evidence rather than a validated clinical comparison.

#### 9.5.1. Benzodiazepines and Non-Benzodiazepine GABA-A Modulators (“Z-Drugs”)

Traditional benzodiazepines and non-benzodiazepine benzodiazepine receptor agonists (“Z-drugs”) primarily act through potentiation of GABA-A receptor signaling. Although effective for short-term insomnia management, these agents may adversely affect gut motility, alter microbiome composition, impair sleep architecture, and potentially reduce glymphatic clearance efficiency [196,292,293,294,295,296]. In older adults, chronic use has been associated with cognitive impairment, increased fall risk, and concerns regarding dementia risk, leading many of these medications to be classified as potentially inappropriate in geriatric populations [297].

#### 9.5.2. Dual Orexin Receptor Antagonists (DORAs)

In contrast, newer sleep therapeutics may better preserve physiological sleep architecture. Dual orexin receptor antagonists (DORAs), including suvorexant, lemborexant, and daridorexant, maintain slow-wave and REM sleep while targeting wake-promoting orexin signaling [298,299,300]. Emerging evidence suggests that these agents may favorably influence Alzheimer’s-disease-related biomarkers, including reductions in cerebrospinal fluid amyloid-β and phosphorylated tau levels [301,302,303]. Some studies additionally suggest beneficial effects on neuroinflammation and circadian regulation, although their role in Parkinson’s disease remains less clear [304].

#### 9.5.3. Melatonin Receptor Agonists

As also discussed previously in Section 9.2.1, melatonin receptor agonists and exogenous melatonin represent another promising class because they target pathways discussed throughout this review [49,116,305]. Beyond improving circadian alignment, and based largely on preclinical animal models, melatonin may influence gut microbial composition and intestinal barrier integrity, attenuate systemic and neuroinflammatory signaling, and regulate the circadian polarization of AQP4 at the glymphatic interface, driving perivascular clearance [305,306,307,308,309]. Preclinical studies suggest that melatonin-mediated remodeling of the gut microbiome may contribute to reductions in microglial activation and neurodegenerative pathology, making this class particularly relevant to the microbiota–gut–brain axis [133,307,310].

#### 9.5.4. Histamine H1 Receptor Antagonists

Low-dose doxepin, a selective histamine H1 receptor antagonist approved for sleep maintenance insomnia, occupies an intermediate position within this framework. Unlike benzodiazepines and Z-drugs, low-dose doxepin largely preserves sleep architecture and lacks significant anticholinergic activity, although evidence regarding its effects on gut microbial composition, glymphatic function, and long-term neurodegenerative outcomes remains limited [297,311,312].

These observations suggest that sleep medications should not be viewed solely as symptomatic treatments for insomnia. Rather, their effects on sleep architecture, gut microbial ecology, autonomic signaling, neuroinflammation, and glymphatic clearance may have important implications for long-term neurological health. Future studies should therefore evaluate hypnotic agents both according to their effects on sleep quality but also by their influence on biomarkers of gut–brain communication and neurodegenerative disease progression. Although many therapeutic approaches remain experimental, several lifestyle factors influence the same biological pathways and may provide accessible strategies for preserving gut–brain–sleep axis function.

### 9.6. Lifestyle Factors Influencing the Gut–Brain–Sleep Axis

The preservation of neurological health across the lifespan is increasingly understood to depend on modifiable lifestyle factors that operate through interconnected biological systems. Emerging research highlights that the gut–brain axis, the glymphatic system, and the gut microbiome can have an add-on effect in mediating neurodegeneration risk, and that behavioral choices meaningfully influence each of these systems.

#### 9.6.1. Diet and Nutritional Interventions

When considering non-therapeutic factors in gut–brain axis health, diet is one of the most powerful lifestyle modifiers available. A Mediterranean dietary pattern rich in fiber, polyphenols, and omega-3 polyunsaturated fatty acids (n-3 PUFAs) is associated with reduced risk of both Parkinson’s and Alzheimer’s disease [313,314]. These effects can be partly attributed to microbiome modulation [315]. High fiber intake promotes the production of SCFAs, reinforcing the anti-inflammatory mechanisms and barrier protective effects described in Section 4.3 and Section 6.3 [315].

Calorie restriction has also demonstrated the capacity to slow aging, enhance metabolic health and stimulate autophagy, mechanisms that potentially support both glymphatic clearance and microbial diversity [316,317,318,319,320,321]. Intermittent fasting protocols, which may be more accessible, represent a promising behavioral intervention that may optimize metabolic flux and promote systemic autophagy without the restrictive burdens of chronic caloric deficit [322]. Intermittent fasting can also affect sleep architecture and enhance circadian stability, further modulating the gut microbiome-derived metabolites that are essential for metabolic regulation and neuroprotection [323]. Chrono-nutrition has been gaining attention in recent years, including focus on its potential in affecting sleep health, however more research is needed to better understand optimal timing for nutrient intake to align with circadian rhythms and maximize the restorative capacity of the gut–brain axis [324]. Diets such as the MIND diet have been associated with cognitive benefits as part of multidomain lifestyle interventions, and also with improved sleep quality [313,325,326,327,328].

Omega-3 polyunsaturated fatty acids (n-3 PUFAs) like those found in fish oil have an anti-inflammatory and antioxidant effect and help maintain BBB integrity [329]. In preclinical models, n-3 PUFAs have been found to also protect against loss of AQP4 polarization on astrocytic endfeet, which is associated with preserved glymphatic integrity allowing clearance of amyloid-beta from the brain [330,331,332]. They may help regulate sleep efficiency as well; however, more research is needed to better clarify their use in humans in a tailored medicine approach [333].

#### 9.6.2. Sleep Optimization

Maintenance of proper sleep quality is consequential as mentioned above and helps modulate the risk for neurodegenerative processes. As previously discussed, glymphatic clearance operates predominantly during slow-wave sleep. Thus, chronically disrupted sleep, which is increasingly prevalent in aging populations, impairs this clearance mechanism and accelerates the accumulation of neurotoxic proteins. Sleeping in the lateral position also appears to further optimize glymphatic transport compared to supine or prone orientations [330]. This is often a small, but very manageable, lifestyle modification for most patients. Optimization of all sleep pathologies, like insomnia and sleep apnea, is key. The choice of hypnotic in case of insomnia should be carefully weighted as medications may affect both sleep waves and glymphatic system [196] and the gut microbiome [334]. More longitudinal attention is needed during clinical trials beyond the pure hypnotic effect, particularly in an aging population.

#### 9.6.3. Physical Activity

Regular physical exercise benefits all three systems simultaneously. Exercise increases microbiota diversity [315], promotes AQP4 polarization to enhance glymphatic clearance, and reduces amyloid-beta accumulation in animal models [335]. It also preserves beneficial SCFA-producing organisms, including *Faecalibacterium* and *Lachnospiraceae*, which are often reported as depleted in individuals with neurodegenerative diseases [315]. Exercise’s role in improving global cognitive function in humans is indirectly supported by multi-domain lifestyle trials, such as FINGER and its US replicate, the US-POINTER trial, in which exercise was combined with a brain-healthy diet, cognitive stimulation activities and vascular risk factor monitoring [313,327,328].

#### 9.6.4. Stress Management and Mind–Body Interventions

Chronic psychological stress, conversely, impairs glymphatic influx and efflux through glucocorticoid-mediated reductions in AQP4-bearing astrocytes [330], while simultaneously promoting gut dysbiosis and systemic inflammation [336]. Integrating mind–body therapies like yoga or meditation can further complement these physiological interventions by attenuating chronic stress responses, which are known to trigger systemic dysbiosis and exacerbate inflammatory cascades [33,337].

The current evidence supports a model in which diet, sleep, exercise, and stress management converge upon common biological pathways involving the gut microbiome, neuroimmune signaling, and glymphatic clearance. These lifestyle factors represent accessible, low-risk interventions that may simultaneously target multiple drivers of neurodegenerative disease. Future studies integrating microbiome analyses, sleep metrics, metabolomics, neuroimaging, and cognitive outcomes will be essential for determining how combinations of lifestyle interventions can be personalized to maximize long-term neurological health and resilience during aging.

## 10. Conclusions and Perspectives

The gut–brain axis, sleep, and neurodegeneration are increasingly being recognized as an interconnected system, though as discussed throughout this review, most of the specific connections in this proposed network are supported by preclinical or associative evidence rather than properly tested human evidence [130,338].

Throughout this review, we have highlighted multiple opportunities for intervention across the gut–brain–sleep axis, raising the possibility of more effective preventive and therapeutic approaches. This includes the potential for targeted interventions at multiple levels including dietary, lifestyle, and non-invasive interventions, including prebiotic and probiotic supplementation, microbiome-modulating dietary patterns, and non-invasive neuromodulation techniques, such as vagus nerve stimulation.

Despite these promising advances, several important limitations remain that are typical of a narrative review. Much of the mechanistic evidence is from preclinical animal models that may not fully reflect the reality and complexity of human sleep and neurodegeneration. For example, rodent model research is conducted under tightly controlled nutritional and circadian rhythms, which do not reflect the actual human reality. While the estrobolome and bile acid signaling represent promising therapeutic targets, the fluctuations and diversity of the human microbiome, as well as our limited understanding of its heterogeneity in composition, are still significant challenges that will need to be addressed further. Finally, this approach focuses more on the body-first paradigm, but evidence suggests that a significant number of individuals will follow a more brain-first course. More research is needed to better understand the biological and genetic background of each subtype and how each responds to estrobolome and exposome modifications, allowing for better stratification and homogenization in research and clinical trial cohorts that more accurately depict the underlying biology and pathology. Future clinical investigations should prioritize longitudinal studies over observational data to better delineate the precise temporal dynamics of these systemic interactions, potentially identifying biomarkers that predict individual profile, as well as their potential responsiveness to these integrated, lifestyle-based and therapeutic intervention strategies.

An additional limitation concerns confounding and the possibility of reverse causality. Several of the factors discussed throughout this review, including age and dysbiosis (Section 7.1), sex hormones and menopausal status (Section 7.2, Section 7.3 and Section 7.4), diet and other lifestyle behaviors (Section 9.6), obstructive sleep apnea (Section 2), hypnotic and other medication use (Section 9.5) and autonomic dysfunction in neurodegenerative disease (Section 3), may independently influence both sleep physiology and gut microbiome composition. Because most of the underlying evidence is cross-sectional or derived from animal models, it is often not possible to determine whether the microbiome or sleep alterations are causal contributors to neurodegeneration, secondary consequences of shared upstream factors, or downstream effects of early, subclinical neurodegenerative processes themselves. Disentangling these possibilities will require longitudinal human studies that explicitly account for these confounders.

Building on the gaps identified throughout this review, specific and experimentally testable research priorities include the following: (1) determining whether microbiome-targeted interventions (e.g., FMT, SCFA supplementation) alter glymphatic clearance, as measured by DTI-ALPS, in longitudinal human cohorts (Section 6.3); (2) testing whether vagus nerve stimulation or vagotomy measurably change biomarkers of alpha synuclein propagation in prospective human cohorts, to move beyond current epidemiological associations (Section 4.1); (3) determining whether estrobolome-targeted interventions (e.g., beta-glucuronidase-modulating probiotics) alter amyloid or neuroinflammatory biomarkers in peri- and post-menopausal women (Section 7.3, Section 7.4 and Section 9.2.2); (4) conducting longitudinal studies that stratify patients by brain-first versus body-first phenotype (Section 3) to determine whether this distinction predicts differential responses to gut-directed versus centrally directed therapies; and (5) directly testing, in controlled human trials, whether bile acid modulation improves sleep architecture and glymphatic clearance, rather than relying on the current preclinical and correlational evidence (Section 4.4 and Section 9.2.3).

A major framework shift is currently emerging in neurodegenerative disease research and expanding our vision beyond brain-only diseases to consider them as more systemically based diseases, possibly with variable progression leading to different phenotypes integrated to the clinical and genetic background, and dynamic interactions among sleep physiology, gut microbial ecosystems, immune signaling, endocrine regulation, autonomic function, and glymphatic clearance. Understanding these interconnected systems may enable earlier biomarker discovery, improved patient stratification, and the development of multi-target interventions that preserve cognitive resilience across the lifespan [339,340].

## Figures and Tables

**Table 1:** Major pathways linking sleep, the gut microbiome, and neurodegeneration.

Pathway	Key Mediators	Role in Sleep Regulation	Role in Neurodegeneration	Potential Biomarkers	Therapeutic Opportunities	Evidence
Glymphatic Clearance System	AQP4, norepinephrine, CSF–ISF exchange, meningeal lymphatics	Peaks during slow-wave sleep; impaired by sleep loss/circadian disruption	Reduced Aβ/tau/α-synuclein clearance promotes neurodegeneration	DTI-ALPS, AQP4 polymorphisms, sleep EEG metrics	Sleep optimization, AQP4-targeted therapies, VEGF-C, photobiomodulation	Moderate Mice and human studies
Vagus Nerve Signaling	α-synuclein, cytokines, microbial metabolites, vagal afferent activity	Modulates brainstem arousal circuits; linked to RBD and sleep fragmentation	Bidirectional gut–brain pathology spread; implicated in body-first PD	Heart rate variability, vagal tone measures, autonomic testing, vagal imaging	Vagus nerve stimulation (VNS), transcutaneous VNS (tVNS), autonomic modulation	Low–moderate Mice models; human epidemiological studies
Immune-Inflammatory Signaling	IL-6, TNF-α, IL-1β, LPS, NLRP3 inflammasome	Regulates sleep–wake behavior; chronic inflammation disrupts sleep/circadian rhythm	Drives microglial activation, BBB dysfunction, amyloid/tau pathology	Plasma cytokines, CRP, LPS, fecal calprotectin, zonulin	Anti-inflammatory therapies, microbiome modulation, dietary interventions	Low–moderate Mice models, human studies
Tryptophan– Serotonin– Melatonin Axis	Tryptophan metabolites, serotonin, melatonin, kynurenines	Regulates circadian timing, sleep onset/maintenance	Dysregulation drives neuroinflammation, oxidative stress, cognitive decline	Melatonin levels, kynurenine/tryptophan ratio, serotonin metabolites	Melatonin supplementation, circadian interventions, microbiome-targeted therapies	Low–moderate Mice models, human physiology
Estrobolome-Hormonal Signaling	β-glucuronidase, estrogen metabolites, FSH	Menopausal hormone changes drive insomnia, sleep fragmentation	May contribute to female-biased AD risk via estrogen/microglial signaling	β-glucuronidase activity, estrogen metabolites, hormone levels	Targeted probiotics, hormone replacement, SERMs	Low–moderate Mice models, human cross-sectional studies
Gut Barrier Integrity (“Leaky Gut”)	Tight junction proteins, zonulin, LPS, microbial amyloids	Sleep loss increases gut permeability; barrier dysfunction worsens sleep via inflammation	Endotoxin/microbial product translocation promotes neuroinflammation, protein aggregation	Zonulin, fecal calprotectin, LPS, intestinal permeability assays	Dietary interventions, probiotics, SCFA restoration, anti-inflammatory therapies	Low–moderate Mice models, human observational/biomarker studies
Bile Acid Signaling	Primary and secondary bile acids, FXR, TGR5 receptors	Influences arousal pathways, circadian regulation, and sleep fragmentation	Alters neuroinflammation, metabolic homeostasis, BBB integrity, and cognitive function	Serum and stool bile acid profiles	Bile acid receptor agonists, microbiome-directed therapies	Low Mice studies
Microbial Amyloid Signaling	Curli proteins, bacterial amyloids, LPS	Indirect effects through inflammation and sleep disruption	May cross-seed amyloid-β/α-synuclein aggregation, prime microglial inflammation	Curli-producing taxa, microbial metagenomics	Microbiome modulation, anti-inflammatory therapies, barrier restoration	Low Mice models, basic science
Short-Chain Fatty Acids (SCFAs)	Butyrate, acetate, propionate	Regulate circadian gene expression, neurotransmitter production	Support BBB integrity, suppress neuroinflammation, maintain AQP4 polarization	Stool/serum SCFA levels, microbiome profiling	High-fiber diets, prebiotics, probiotics, SCFA supplementation	Low Mice models only

**Table 2:** Emerging biomarkers of the gut–brain–sleep axis.

Domain	Biomarker	Biological Meaning	Clinical Potential	Use Status
Microbiome	Alpha diversity	Dysbiosis	Risk stratification	Research
Metabolites	SCFAs	Anti-inflammatory signaling	Monitoring	Research
Bile acids	Secondary BA profile	Gut–liver–brain signaling	Sleep disorders	Research
Barrier	Zonulin	Intestinal permeability	Leaky gut	Candidate
Barrier	Calprotectin	Gut inflammation	Screening	Clinical/ Candidate
Hormonal	β-glucuronidase activity	Estrobolome function	Menopause/hormonal risk monitoring	Candidate
Imaging	DTI-ALPS	Indirect glymphatic activity	Investigational—early AD	Research
Imaging	TSPO-PET	Neuroinflammation	Disease monitoring	Research
Sleep	PSG metrics	Sleep fragmentation	Risk prediction	Clinical
Autonomic/GI	EGG	Gastric dysrhythmia/ vagal dysfunction	Early PD (prodromal)	Research

**Table 3:** Summary of therapeutic interventions and level of evidence.

Intervention	Primary Target/ Mechanism	Direction of Effect in Humans	Level of Evidence
Multidomain lifestyle interventions	Multiple pathways	Positive	High
Vagus nerve stimulation (VNS/tVNS)	Vagal afferent signaling	Preliminary positive	Moderate
Melatonin receptor agonists	MT1/MT2 receptors	Preliminary Positive	Low–moderate
Probiotics/prebiotics	Microbiome composition	Preliminary positive	Low–moderate
Dual orexin receptor antagonists (DORAs)	OX1R/OX2R signaling	Positive, biomarker level (AD); unclear or negative (PD)	Moderate
Fecal microbiota transplantation (FMT)	Gut microbiome	Exploratory	Low
Microbiome-targeted stem cell and strain specific therapies	Stem cells, Bifidobacterium strains	Not yet tested	Low
Estrobolome-targeted therapy	Estrogen/β-glucuronidase signaling	Not yet tested	Low
Bile acid modulation	FXR/TGR5 signaling	Not yet tested	Low
Glymphatic enhancement	AQP4/CSF-ISF exchange	Not yet tested	Low
Benzodiazepines (BZDs)	GABA-A receptor	Negative	Low–moderate
Non-benzodiazepine GABA-A modulators (Z-drugs)	GABA-A receptor	Negative	Low–moderate
Histamine H1 antagonists (low-dose doxepin)	H1 receptor	Neutral to negative (dose-dependent)	Low

## Data Availability

No new data were created or analyzed in this study. Data sharing is not applicable to this article.
